# Ribonucleotide reductase small subunit M2 is a master driver of aggressive prostate cancer

**DOI:** 10.1002/1878-0261.12706

**Published:** 2020-05-31

**Authors:** Ying Z. Mazzu, Joshua Armenia, Subhiksha Nandakumar, Goutam Chakraborty, Yuki Yoshikawa, Lina E. Jehane, Gwo‐Shu Mary Lee, Mohammad Atiq, Nabeela Khan, Nikolaus Schultz, Philip W. Kantoff

**Affiliations:** ^1^ Department of Medicine Memorial Sloan Kettering Cancer Center New York NY USA; ^2^ Human Oncology and Pathogenesis Program Memorial Sloan Kettering Cancer Center New York NY USA; ^3^ Department of Medical Oncology Dana‐Farber Cancer Institute Boston MA USA; ^4^ Marie‐Josée and Henry R. Kravis Center for Molecular Oncology Memorial Sloan Kettering Cancer Center New York NY USA; ^5^ Department of Epidemiology and Biostatistics Memorial Sloan Kettering Cancer Center New York NY USA

**Keywords:** molecular subtyping, PAM50, PCS subtyping, prostate cancer, RRM2

## Abstract

Although there are molecularly distinct subtypes of prostate cancer, no molecular classification system is used clinically. The ribonucleotide reductase small subunit M2 (*RRM2*) gene plays an oncogenic role in many cancers. Our previous study elucidated comprehensive molecular mechanisms of *RRM2* in prostate cancer (PC). Given the potent functions of *RRM2*, we set out to determine whether the RRM2 signature can be used to identify aggressive subtypes of PC. We applied gene ontology and pathway analysis in RNA‐seq datasets from PC cells overexpressing RRM2. We refined the RRM2 signature by integrating it with two molecular classification systems (PCS and PAM50 subtypes) that define aggressive PC subtypes (PCS1 and luminal B) and correlated signatures with clinical outcomes in six published cohorts comprising 4000 cases of PC. Increased expression of genes in the RRM2 signature was significantly correlated with recurrence, high Gleason score, and lethality of PC. Patients with high *RRM2* levels showed higher PCS1 score, suggesting the aggressive PC feature. Consistently, *RRM2*‐regulated genes were highly enriched in the PCS1 signature from multiple PC cohorts. A simplified RRM2 signature (12 genes) was identified by intersecting the RRM2 signature, PCS1 signature, and the PAM50 classifier. Intriguingly, inhibition of RRM2 specifically targets PCS1 and luminal B genes. Furthermore, 11 genes in the RRM2 signature were correlated with enzalutamide resistance by using a single‐cell RNA‐seq dataset from PC circulating tumor cells. Finally, high expression of *RRM2* was associated with an immunosuppressive tumor‐immune microenvironment in both primary prostate cancer and metastatic prostate cancer using CIBERSORT analysis and LM22, a validated leukocyte gene signature matrix. These data demonstrate that *RRM2* is a driver of aggressive prostate cancer subtypes and contributes to immune escape, suggesting that RRM2 inhibition may be of clinical benefit for patients with PC.

## Graphical abstract

RRM2 is essential for DNA synthesis and repair. We integrated clinical and experimental datasets to generate RRM2 signatures that not only define subtypes with poor outcomes but also predict enzalutamide resistance. RRM2 overexpression is associated with an immunosuppressive tumor‐immune microenvironment. RRM2 signatures may be novel biomarkers for aggressive prostate cancer subtypes, and targeting RRM2 could be a useful therapeutic strategy.

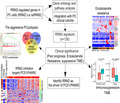

**Abbreviations**
AR: androgen receptor
CIBERSORT: cell‐type identification by estimating relative subsets of RNA transcripts
CRPC: castration‐resistant prostate cancer
CTCs: circulating tumor cells
DFS: disease‐free survival
dNTPs: deoxyribonucleotide triphosphates
EMT: epithelial–mesenchymal transition
ENZ: enzalutamide
FC: fold change
FDR: false discovery rate
GEO: Gene Expression Omnibus
GO: gene ontology
GRID: Genomic Resource Information Database
GSEA: gene set enrichment analysis
GSVA: gene set variation analysis
PCS: prostate cancer subtype
PCS1: prostate cancer subtype 1
PCS2: prostate cancer subtype 2
PCS3: prostate cancer subtype 3
PSA: prostate‐specific antigen
RNA‐seq: RNA sequencing
*RRM2*: ribonucleotide reductase subunit M2
ssGSEA: single‐sample gene set enrichment analysis
SU2C/PCF: Stand Up To Cancer/Prostate Cancer Foundation
TCGA: The Cancer Genome Atlas
TIL: tumor‐infiltrating lymphocyte
TIME: tumor‐immune microenvironment

## 1 Introduction

Prostate cancer is a heterogeneous disease and the third leading cause of cancer death among American men. Clinical decision making has been largely driven by clinical and pathologic variables, such as tumor stage, Gleason score, and serum prostate‐specific antigen (PSA) levels (Falzarano and Magi‐Galluzzi, 2011; Gleason and Mellinger, 1974). Inhibition of androgen receptor (AR) signaling is the mainstay of therapy for recurrent or advanced prostate cancer (Assikis and Simons, 2004) but is limited in its utility because of acquired resistance (Attard *et al*., 2016). There is an unmet clinical need to identify patients with aggressive and drug‐resistant prostate cancer and develop therapies to treat these patients.

Molecular classification has been successfully applied in many cancers and is routinely used to guide treatment decisions (Perou *et al*., 2000). In contrast, molecular subtyping of prostate cancer is based on the underlying genomic alterations and is less established as a determinant of prognosis and guide to treatment. Multiple studies have attempted to establish individual biomarkers or gene expression signatures to predict aggressive cases of prostate cancer (Bibikova *et al*., 2007; Cuzick *et al*., 2011; Glinsky *et al*., 2005; Penney *et al*., 2011), but these studies were limited by the small number of samples analyzed. Recently, You *et al*. reported a novel molecular classification of prostate cancer subtypes (PCS) that was generated from transcriptomic data from more than 4600 prostate cancer specimens. This classification categorizes prostate cancer into three distinct molecular subtypes (PCS1, PCS2, and PCS3) and was validated in ten independent prostate cancer cohorts and several preclinical *in vitro* and *in vivo* prostate cancer models (You *et al*., 2016). The PCS classification system appears useful in distinguishing aggressive disease using both the tumor and blood of patients with prostate cancer. In addition to the PCS signatures, the PAM50 classifier, which was commercially developed as Prosigna to assess breast cancer risk (Nielsen *et al*., 2014), was recently proven to segregate prostate cancer into three subtypes (luminal A, luminal B, and basal) in retrospective and prospective cohorts totaling 3782 samples (Zhao *et al*., 2017).

Both the PCS1 and luminal B signatures can be used to effectively identify cases of prostate cancer with poor prognosis, but treating these patients will require an understanding of the molecular drivers of these subtypes. Although the FOXM1 pathway was recently identified as a key driver of PCS1 tumors (Ketola *et al*., 2017), small molecules targeting transcription factors are difficult to develop, and there are no specific FOXM1 inhibitors for clinical application. Similar to *FOXM1*, ribonucleotide reductase subunit M2 (*RRM2*) is a highly expressed gene in the PCS1 and luminal B signatures. RRM2 maintains the deoxyribonucleotide triphosphate (dNTP) pool to support DNA synthesis and repair (Kumar *et al*., 2011) and is overexpressed in multiple cancers (Grade *et al*., 2011; Kretschmer *et al*., 2011). We previously reported the significant prognostic value of *RRM2* in prostate cancer by analyzing 11 prostate cancer cohorts (Mazzu *et al*., 2019). We elucidated the molecular mechanisms underlying its potent oncogenic function by knocking down or overexpressing RRM2 in multiple prostate cancer cell lines. Additionally, we demonstrated that COH29, an RRM2 inhibitor currently in clinical trials for solid tumors, had efficacy against prostate cancer cells *in vitro* and *in vivo*.

In this study, we further demonstrated that *RRM2* is a druggable driver of PCS1 and luminal B tumors. Bioinformatic analysis revealed that *RRM2*‐regulated genes are highly enriched in PCS1 genes and are significantly correlated with clinical outcomes. Tumors with high expression of *RRM2* have tumor‐infiltrating lymphocyte (TIL) populations consistent with an immunosuppressive microenvironment. Finally, we demonstrated that targeting RRM2 specifically inhibits the expression of genes in the PCS1 and luminal B signatures.

## 2 Materials and methods

### 2.1 Clinical cohort summary

All publicly available prostate cancer cohorts used in this study are summarized in Table 1.

**Table 1:** Details of the prostate cancer clinical cohorts that were used in the study. aCGH, array comparative genomic hybridization; BCR, biochemical recurrence; dbGaP, database of Genotypes and Phenotypes; NCI GDC, National Cancer Institute Genomic Data Commons; OS, overall survival; PRAD, prostate adenocarcinoma; RPPA, reverse‐phase protein array; WES, whole‐exome sequencing.

Cohort name	Benign/normal tissue number	Tumor number	Primary number	Metastasis number	Clinical outcome	Data type	Year	Accession number	Reference
TCGA	0	333	333	0	BCR	WES, RNA‐seq, RPPA	2015	TCGA‐PRAD (NCI GDC Data Portal)	CGA Research Network, TCGA Data Portal
Taylor	29 normal	216	131	19	BCR	aCGH, RNA‐seq	2010	GSE21032 (GEO)	Taylor *et al*. (2010)
SU2C/PCF	0	150	0	150	BCR	WES, RNA‐seq	2015	Phs000915.v1.p1 (dbGaP)	Robinson *et al*. (2015)
Kumar	176 benign	176	22	154	BCR	aCGH, WES, microarray	2016	GSE77930 (GEO)	Kumar *et al*. (2016)
Grasso	28 benign	122	59	35	OS	aCGH, microarray	2012	GSE35988 (GEO)	Grasso *et al*. (2012)
Setlur	0	363	363	0	OS	microarray	2008	GSE8402 (GEO)	Setlur *et al*. (2008)

### 2.2 Cell culture

LNCaP (RRID: CVCL_0395) and PC‐3 (RRID: CVCL_0035) cells were purchased from ATCC (Manassas, VA, USA). C4‐2 (LNCaP C4‐2, RRID: CVCL_4782) cells were obtained from VitroMed (Burlington, NC, USA). As previously described (Mazzu *et al*., 2019), lentiviral vectors encoding *RRM2* were infected in LNCaP and PC‐3 cells, and stable cell lines were generated and maintained using puromycin selection. Efficiency of overexpression was verified by qPCR and western blot. All cells were maintained in media with 10% FBS (Thermo Fisher Scientific, Waltham, MA, USA) supplemented with 2 mm of l‐glutamine (Thermo Fisher Scientific) and 100 U·mL^−1^ penicillin/streptomycin (Thermo Fisher Scientific) at 37 °C in 5% CO_2_. Cell line authentication was performed by human short‐tandem repeat profiling at the Memorial Sloan Kettering Cancer Center Integrated Genomics Operation within the last 3 years. Experiments were performed in mycoplasma‐free cell lines.

### 2.3 Gene silencing and overexpression

SMARTpool siRNAs (Dharmacon, Lafayette, CO, USA) were used for transfection with RNAiMAX (Thermo Fisher Scientific) to knock down target gene expression. For overexpression, cells were transduced with lentiviral vectors encoding *RRM2* and selected by treatment with puromycin as described previously (Mazzu *et al*., 2019). Efficiency of knockdown and overexpression was verified after 2 or 3 days by qPCR and western blot.

### 2.4 RNA sequencing

Total RNA was extracted from cells and analyzed as previously described (Zhang *et al*., 2011). RNA sequencing (RNA‐seq) was performed by 50 million 2 × 50 bp reads at the Memorial Sloan Kettering Cancer Center Integrated Genomics Operation, and data were analyzed in partek flow software (St. Louis, MO, USA). The data are available from GEO (GSE117921–GSE117924).

### 2.5 Bioinformatic analysis of clinical cohorts

Bioinformatic analysis of the clinical cohorts was performed using data obtained from cBioPortal for Cancer Genomics (Gao *et al*., 2013) and Oncomine (Rhodes *et al*., 2004). Heat maps and volcano plots were generated using r version 3.4.3 (https://www.R‐project.org). Pathway analysis from RNA‐seq data was performed using gene set enrichment analysis (GSEA) and ToppGene (Chen *et al*., 2009; Subramanian *et al*., 2005).

The enrichments function in cBioPortal was used to identify genes with expression that was significantly correlated with *RRM2* overexpression (*RRM2*: EXP > 1.5, *z*‐score) in prostate cancer clinical cohorts. Only genes with expression that positively correlated with *RRM2* levels [*R* > 0.5, false discovery rate (FDR) < 0.05] from published prostate cancer cohorts [The Cancer Genome Atlas (TCGA), Kumar, and Stand Up To Cancer/Prostate Cancer Foundation (SU2C/PCF)] were selected (Kumar *et al*., 2016; Network, 2015; Robinson *et al*., 2015). These genes (*n* = 626) were intersected with gene expression data from the PC‐3 and LNCaP cell lines with stable *RRM2* overexpression to develop the RRM2 signature.

Prostate cancer subtype scores were calculated with gene set variation analysis (GSVA) using single‐sample GSEA (ssGSEA) (Barbie *et al*., 2009). Briefly, PCS signature scores were defined by the quantification of the composite expression of each gene in the signature in each sample. We computed a *z*‐score for the expression of each gene in each sample by subtracting the pooled mean from the RNA‐seq expression values and dividing by the pooled standard deviation. The overall survival analysis with the 12‐gene signature was performed using KM plotter (www.kmplot.com/mirpower) (Lanczky *et al*., 2016).

### 2.6 TIL maps and cell‐type identification by estimating relative subsets of RNA transcripts analysis

In each cohort, samples were categorized as *RRM2* high (upper quantile) or low (lower quantile) based on mRNA expression. The fraction of TILs in TCGA cases was determined with a machine‐learning algorithm that uses digital hematoxylin and eosin (H&E) slides (Saltz *et al*., 2018). The abundance of immune cell fractions in each sample was determined using cell‐type identification by estimating relative subsets of RNA transcripts (CIBERSORT) and LM22, a validated leukocyte gene signature matrix (Newman *et al*., 2015).

### 2.7 Statistical analysis

Results are reported as mean ± standard deviation. Comparisons between groups were performed using an unpaired two‐sided Student's *t*‐test or Wilcoxon rank‐sum test (*P* < 0.05 was considered significant). Disease‐free survival (DFS) was examined using the Kaplan–Meier method. Patients were divided into two groups (upper and lower quartile based on *RRM2* expression or RRM2 signature score), and Kaplan–Meier curves were generated for each group. The log‐rank test was used to determine significance. Cox proportional hazard regression was performed, adjusting for clinical and demographic factors. The significance of the correlation between gene expression and enzalutamide resistance was analyzed by Fisher's exact test. The significance of the differences in the abundance of immune cell types between groups was determined using Wilcoxon's rank‐sum test with Benjamini–Hochberg correction. Statistical analysis was completed using r version 3.4.3 (https://www.R‐project.org).

### 2.8 Data accessibility

RNA‐seq data are available from the Gene Expression Omnibus (GEO: GSE117921, GEO: GSE117922, GEO: GSE117923, and GEO: GSE117924).

## 3 Results

### 3.1 Defining the RRM2 signature and its clinical relevance in prostate cancer

Our prior study reported the potent oncogenic activity and clinical significance of *RRM2* in prostate cancer (Mazzu *et al*., 2019). Although we demonstrated that there was a significant correlation between increased *RRM2* levels and poor clinical outcomes, we believed that the prognostic value of *RRM2* had been underestimated because RRM2 expression is strictly regulated during the cell cycle, with levels peaking during S‐phase, followed by rapid degradation (Chabes and Thelander, 2000). However, its potent oncogenic activity is maintained to support tumor survival and progression (Fujita *et al*., 2010; Lee *et al*., 2014; Su *et al*., 2014). We hypothesized that an RRM2 signature would further elucidate the function of RRM2. To modulate RRM2 activity in prostate cancer cells, we developed two prostate cancer cell lines with stable overexpression of *RRM2* (PC‐3‐*RRM2* and LNCaP‐*RRM2*) and used siRNA and COH29, a small molecule inhibitor of RRM2 (Mazzu *et al*., 2019). Using these cellular models, we were able to explore the transcriptomic changes induced by RRM2, define the downstream mechanisms through which RRM2 functions, and identify an RRM2 signature.

**Fig. 1:**
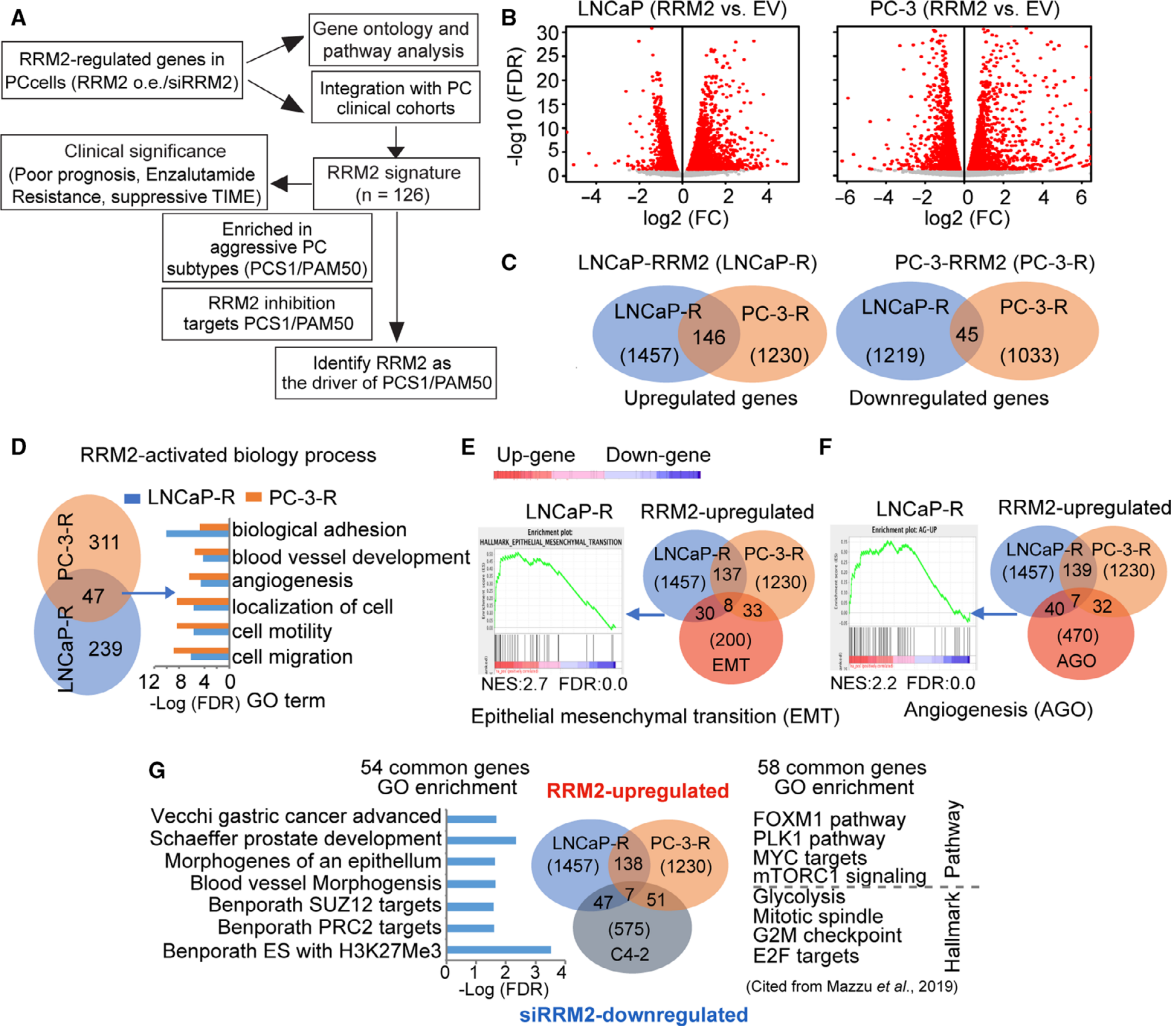
*RRM2* function is disease‐state‐specific. (A) Schematic of the experimental design. As previously reported (Mazzu *et al*., 2019), transcriptomic changes induced by *RRM2* overexpression or inhibition (FDR < 0.05, −1.5 > FC > 1.5) from cellular models were integrated with prostate cancer clinical cohorts to generate an RRM2 signature. Here, we applied the PCS and PAM50 gene sets to further characterize the signature. (B) Volcano plots show transcriptomic changes induced by *RRM2* overexpression in LNCaP (left) and PC‐3 (right) cells. (C) Venn diagrams show the overlap of genes upregulated (left) and downregulated (right) with *RRM2* overexpression in LNCaP and PC‐3 cells (FDR < 0.05, −1.5 > FC > 1.5). (D) Venn diagram (left) depicts the overlap of GO analysis of genes upregulated by *RRM2* overexpression. Bar graphs (right) show common biological processes activated in 2 cell lines with *RRM2* overexpression. (E) Enrichment of *RRM2*‐upregulated genes from LNCaP in EMT and (F) angiogenesis. GSEA results are from LNCaP cells, and Venn diagrams show the overlap between pathway genes and genes upregulated in LNCaP and PC‐3 cells that overexpress *RRM2*. (G) GO enrichment of common genes deregulated in LNCaP‐*RRM2*/C4‐2‐si*RRM2* (left) and PC‐3‐*RRM2*/C4‐2‐si*RRM2* (right).

To uncover downstream pathways, genes deregulated with manipulation of *RRM2* [FDR < 0.05, −1.5 > fold change (FC) > 1.5] were subjected to gene ontology (GO) analysis (Fig. 1A). To identify an RRM2 signature, these genes were also compared to the genes with expression that correlated with *RRM2* levels in prostate cancer clinical cohorts. The clinical significance of the RRM2 signature was evaluated in multiple prostate cancer cohorts. To determine whether *RRM2* is a driver of PCSs with poor prognosis, the signatures of two well‐established prostate cancer classifications (PCS and PAM50) were compared to the RRM2 signature.

### 3.2 *RRM2* function is disease‐state‐specific in prostate cancer

We have previously analyzed the transcriptomic changes in PC‐3 cells, an AR‐negative cell line, that overexpress *RRM2* (PC‐3‐*RRM2*); we used this as a castration‐resistant cellular model (Mazzu *et al*., 2019). To compare *RRM2* function in different disease states, we performed RNA‐seq of LNCaP cells, an AR‐positive cell line, that overexpress *RRM2* (LNCaP‐*RRM2*). We confirmed overexpression of *RRM2* in both PC‐3 and LNCaP cells in our previous study (Mazzu *et al*., 2019). In both stable cell lines, a similar number of genes were deregulated by *RRM2* overexpression (Fig. 1B). Among the 1230 PC‐3 and 1457 LNCaP upregulated genes, there were 146 genes that were upregulated in both cell lines. Only 45 downregulated genes were shared among the 1033 PC‐3 and 1219 LNCaP downregulated genes in either cell line (Fig. 1C). Overall, less than 10% of genes were regulated by *RRM2* in both LNCaP and PC‐3 cells, indicating the underlying function of *RRM2* may be disease‐state‐specific, as these two prostate cancer cell lines may represent different disease states because of their AR status.

Given the previously reported strong oncogenic role of *RRM2* in prostate cancer (Mazzu *et al*., 2019), we performed GO analysis on the *RRM2*‐upregulated genes in LNCaP and PC‐3 cells and found that 47 biological processes were activated in both cell lines (Fig. 1D). The top six were related to tumor metastasis, which is consistent with our prior report of *RRM2*‐induced epithelial–mesenchymal transition (EMT) phenotypes in both cell lines (Mazzu *et al*., 2019). Unlike GO analysis, GSEA provides enrichment scores that signify the enrichment of the specific gene set. GSEA demonstrated that *RRM2*‐upregulated genes in LNCaP cells were significantly enriched in EMT and angiogenesis gene sets (Fig. 1E,F), which is similar to the phenotype we previously reported in PC‐3‐*RRM2* cells (Mazzu *et al*., 2019). Surprisingly, only eight of the 38 enriched genes in the EMT gene set and only seven of the 47 enriched genes in the angiogenesis gene set are shared by the two cell lines, suggesting that *RRM2* regulates both pathways in LNCaP and PC‐3 cells through distinct gene sets.

To further understand the molecular mechanisms regulated by *RRM2*, we integrated transcriptomic datasets from si*RRM2*‐treated C4‐2 cells (C4‐2‐si*RRM2*), LNCaP‐*RRM2*, and PC‐3‐*RRM2*. Changes in *RRM2* expression levels in these cell lines were shown in our prior study (Mazzu *et al*., 2019). Previously, our ToppGene analysis revealed that the 58 common genes that were upregulated in PC‐3‐*RRM2* and downregulated in C4‐2‐si*RRM2* were significantly enriched in oncogenic pathways and cancer hallmarks (Fig. 1G) (Mazzu *et al*., 2019). The 54 shared genes between LNCaP‐*RRM2* and C4‐2‐si*RRM2* cells were enriched in gene sets related to prostate development, gastric cancer progression, angiogenesis, and H3K27me3 (Fig. 1G). Only seven genes were shared between the 58 upregulated genes in PC‐3‐*RRM2* and the 54 upregulated genes in LNCaP‐*RRM2*. These results support the hypothesis that *RRM2* may play a similar oncogenic role in PC‐3 and LNCaP cells by regulating distinct gene sets in different biological contexts.

### 3.3 Clinical relevance of the RRM2 signature

**Fig. 2:**
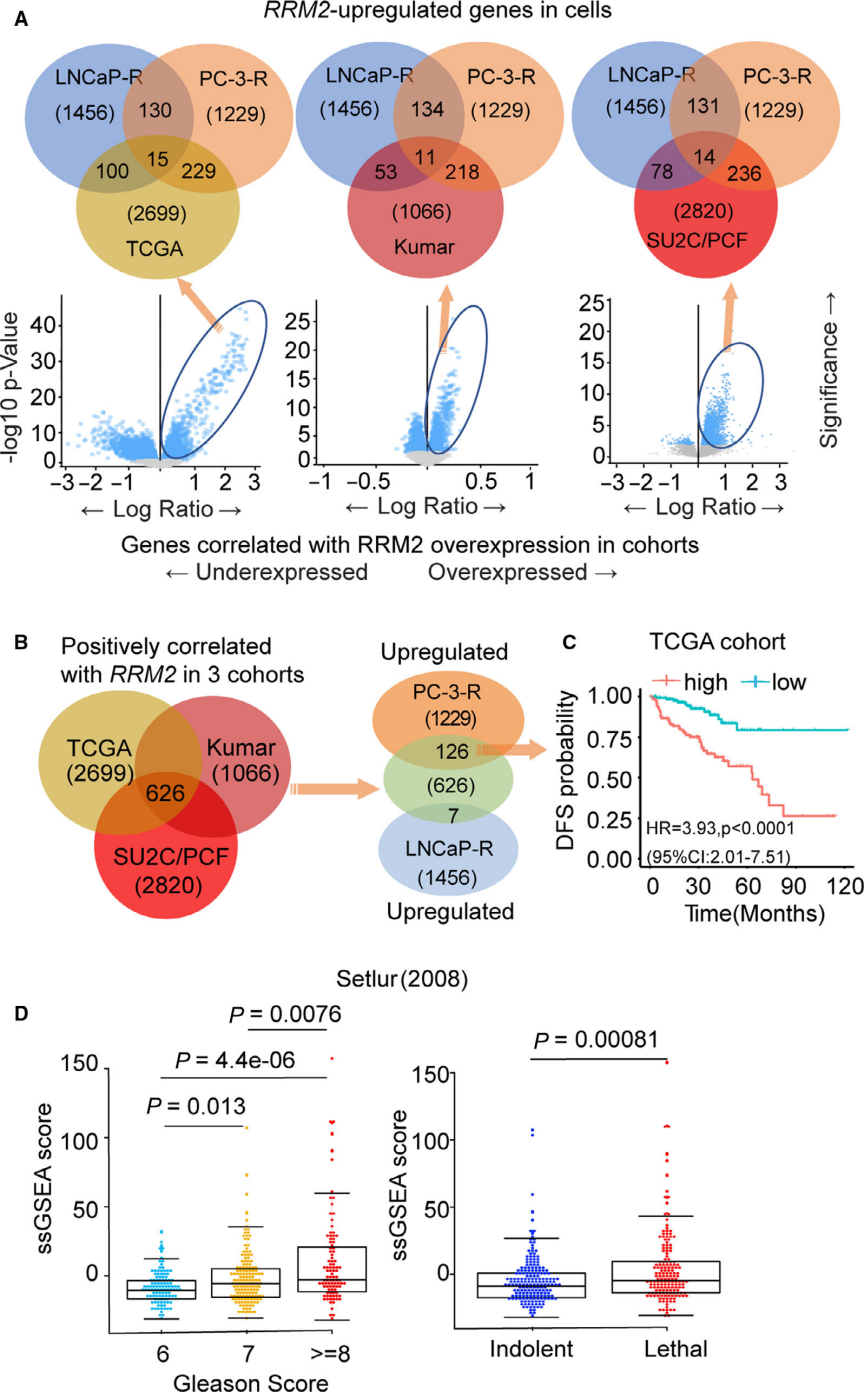
Integration of prostate cancer cell line transcriptomic data with clinical outcomes. (A) Venn diagrams (top) depicting the overlap of genes with expression that positively correlated with *RRM2* levels in TCGA (left), Kumar (middle), and SU2C/PCF (right) cohorts with upregulated genes in LNCaP‐*RRM2* or PC‐3‐*RRM2* cells. Below, plots show the genes with expression that correlates with *RRM2* expression level in each prostate cancer cohort. (B) RRM2 signature: The 626 genes with expression that correlated with *RRM2* levels in the three clinical cohorts (left) were compared with genes upregulated in PC‐3‐*RRM2* or LNCaP‐*RRM2* (right) to identify RRM2 signature (126 genes). (C) Clinical significance of expression of the 126‐gene RRM2 signature in the TCGA cohort. Samples were ranked based on expression of the 126‐gene RRM2 signature, and Kaplan–Meier curves were used to estimate survival differences between patients in the top and bottom 25th percentiles of expression. The log‐rank test was calculated to determine significance. Cox proportional hazard regression was performed, adjusting for clinical and demographic factors. (D) Association between RRM2 signature (126 genes) level with Gleason score (left) and lethality (right) in the Setlur cohort. Comparisons between groups were performed using Wilcoxon's rank‐sum test.

To validate *RRM2*‐regulated genes in prostate cancer clinical samples, we compared genes upregulated in cells that overexpress *RRM2* to those with expression that positively correlated with *RRM2* levels in the TCGA (localized prostate cancer), Kumar [metastatic castration‐resistant prostate cancer (CRPC)], and SU2C/PCF (metastatic CRPC) cohorts (Kumar *et al*., 2016; Network, 2015; Robinson *et al*., 2015) (Fig. 2A). There were approximately 2000–3000 genes with expression that positively correlated with *RRM2* levels in each of the three cohorts. Among these genes, more were upregulated in PC‐3‐*RRM2* (< 250) than in LNCaP‐*RRM2* (< 116, Fig. 2A). When we compared genes from the three cohorts with the genes identified in the cell lines that overexpress *RRM2*, there were 126 genes in PC‐3‐*RRM2* and only seven genes in LNCaP‐*RRM2* that were shared with the clinical cohorts (Fig. 2B and Table S1). Using ssGSEA, we previously reported (Mazzu *et al*., 2019) that the expression of 126 genes was highly correlated with poor DFS in the Taylor cohort (Taylor *et al*., 2010). Here, we confirmed this result in the TCGA cohort (Fig. 2C) and found that increased expression of the 126‐gene signature was significantly correlated with higher Gleason score and lethal disease in the Setlur cohort (Fig. 2D), which has long‐term outcome data (Setlur *et al*., 2008).

### 3.4 High *RRM2* expression is correlated with the poor prognosis prostate cancer subtype PCS1

**Fig. 3:**
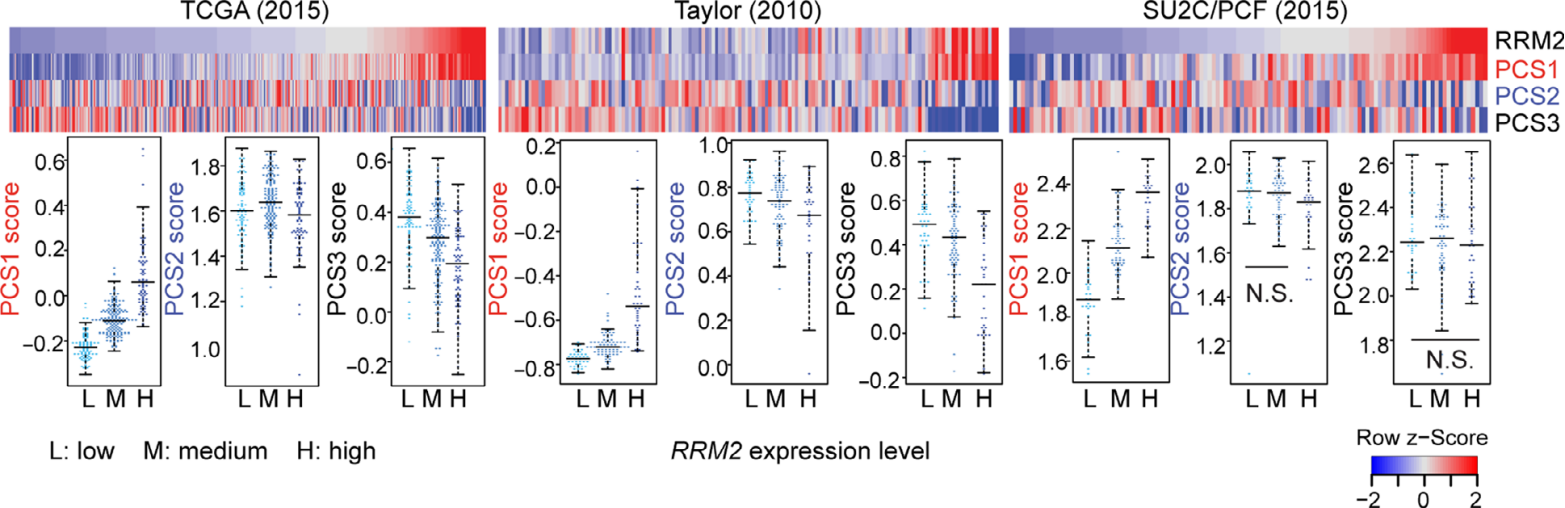
*RRM2* levels are highly correlated with PCS1 gene expression. Correlation of *RRM2* level with PCS gene expression in TCGA (left), Taylor (middle), and SU2C/PCF (right) cohorts. Each individual patient sample is indicated by a single column (top plot) and a single dot (bottom plot). PCS scores were calculated with GSVA using the ssGSEA method, and the values were compared to *RRM2* mRNA expression levels divided by quantiles. The differences between pairs are statistically significant except for those labeled as N.S. (not significant). Significance was determined using Wilcoxon's rank‐sum test.

Among the three prostate cancer subtypes (PCS1–PCS3), PCS1 is the most aggressive and lethal, and PCS1 tumors progress more rapidly to metastatic disease than PCS2 or PCS3 tumors (You *et al*., 2016). The FOXM1 pathway was recently reported as the master regulator of the PCS1 subtype (Ketola *et al*., 2017). We previously reported that *RRM2* is not only a target of FOXM1 but also regulates the FOXM1 pathway (Mazzu *et al*., 2019). Furthermore, *RRM2* is one of the most highly expressed genes in the PCS1 signature. To test our hypothesis that overexpression of *RRM2* could contribute to the development of PCS1 tumors, ssGSEA was performed to determine the correlation between *RRM2* expression level and PCS score in multiple prostate cancer cohorts. In each patient sample, scores of PCS1, PCS2, and PCS3 gene expression were calculated using ssGSEA. In these analyses, *RRM2* was removed from the PCS1 signature to avoid a false‐positive correlation. There was a significant association between high *RRM2* expression and high PCS1 score and low PCS3 score in patient samples in the TCGA and Taylor cohorts (Fig. 3). Intriguingly, the strong correlation between *RRM2* expression level and PCS1 score was also seen in the SU2C/PCF cohort in which all samples are of metastatic CRPC (and *RRM2* expression levels are already high), suggesting that *RRM2* is not only associated with an aggressive PCS but may also regulate multiple key PCS1 genes.

### 3.5 *RRM2* may be a driver of PCS1 tumors

**Fig. 4:**
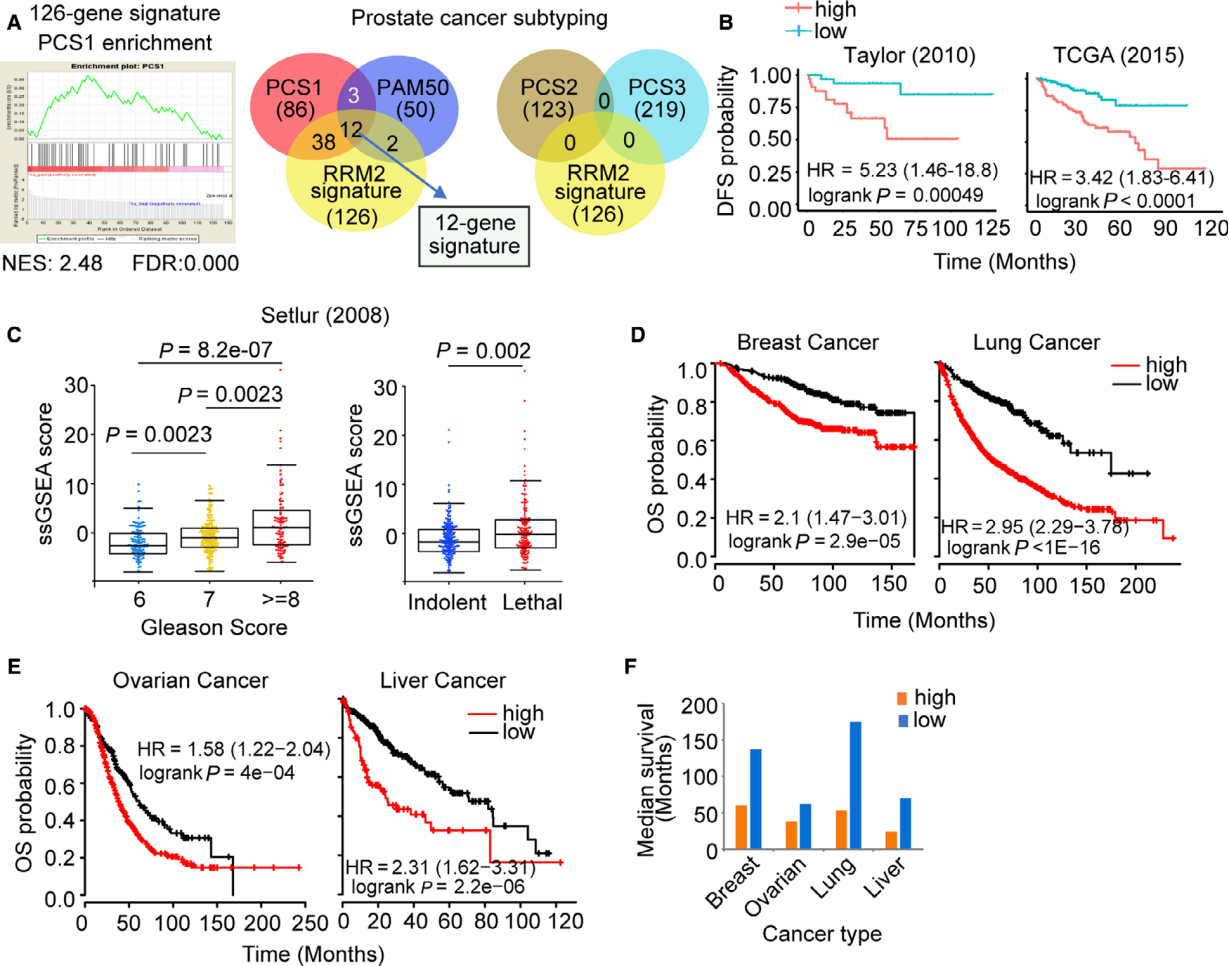
Clinical significance of the 12‐gene RRM2 subsignature. (A) GSEA plot (left) shows high enrichment of PCS1 genes in the RRM2 signature. Venn diagrams (right) depict the overlap between genes in the RRM2 signature with PCS1 and PAM50 genes (left) and PCS2 and PCS3 genes (right). The 12 genes shared by PCS1, PAM50, and RRM2 signature comprise the 12‐gene subsignature. (B) Correlation of expression of the 12‐gene signature with DFS in the Taylor (left) and TCGA (right) cohorts. (C) Correlation of the 12‐gene signature ssGSEA score with Gleason score (left) and lethality (right) in the Setlur cohort. (D) Correlation between 12‐gene signature expression and probability of overall survival (OS) was analyzed in breast and lung cancer and (E) ovarian and liver cancer. Samples were ranked based on expression of the 12‐gene subsignature, and Kaplan–Meier curves were used to estimate survival differences between patients in the top and bottom 25th percentiles of expression. The log‐rank test was calculated to determine significance. Cox proportional hazard regression was performed, adjusting for clinical and demographic factors. Significance was determined using Wilcoxon's rank‐sum test. (F) Median survival time was compared between cases with low (blue) or high (orange) expression of the 12‐gene panel.

Because tumors with high *RRM2* expression have high PCS1 scores, we assessed whether *RRM2*‐regulated genes correlated with PCS signatures. GSEA demonstrated that the 126 *RRM2*‐regulated genes (Fig. 2B) were highly enriched in PCS1 genes (Fig. 4A). Fifty (40%) of the 126 genes overlapped with the 86 PCS1 genes (Fig. 4A and Table S1). Additionally, the PAM50 classifier, which is used in determining breast cancer prognosis, has also been reported to consistently segregate prostate cancer into luminal and basal subtypes that correlate with clinical outcome (Zhao *et al*., 2017). Interestingly, all the overlapping genes in the PCS1 signature and PAM50 classifier are luminal B genes (Table S1). Fourteen genes in the 126‐gene signature overlap with the 50 genes of the PAM50 signature. Among them, 12 genes were shared with both PCS1 and PAM50 genes (Fig. 4A and Table S1). The 126‐gene signature did not share any genes with the PCS2 and PCS3 signatures (Fig. 4A), demonstrating that the signature is predictive of the aggressive subtype of prostate cancer.

We have shown that high expression of the 126‐gene signature is associated with higher Gleason score and shorter patient survival (Fig. 2C,D) (Mazzu *et al*., 2019). Similarly, we found that high levels of the 12‐gene RRM2/PCS1 subsignature were associated with a significant decrease in DFS in the TCGA and Taylor cohorts (Fig. 4B). High expression of the 12 genes was also associated with increased Gleason score and lethality in the Setlur cohort (Fig. 4C). The oncogenic function of *RRM2* has been confirmed in breast, ovarian, lung, and liver cancers, and we assessed whether the 12‐gene signature was associated with poor outcomes in these tumors (Aird *et al*., 2014; Shah *et al*., 2014; Xu *et al*., 2008). High expression of the signature was significantly correlated with worse overall survival in all four cancer types (Fig. 4D,E), with 1.4‐ to 3.3‐fold shorter median survival (Fig. 4F). Altogether, these data suggest that the 12‐gene panel is the core set of genes downstream of *RRM2* that control tumor progression and affect clinical outcomes in prostate cancer and tumors of other cellular origins.

### 3.6 Inhibition of RRM2 activity specifically targets aggressive prostate cancer subtypes

**Fig. 5:**
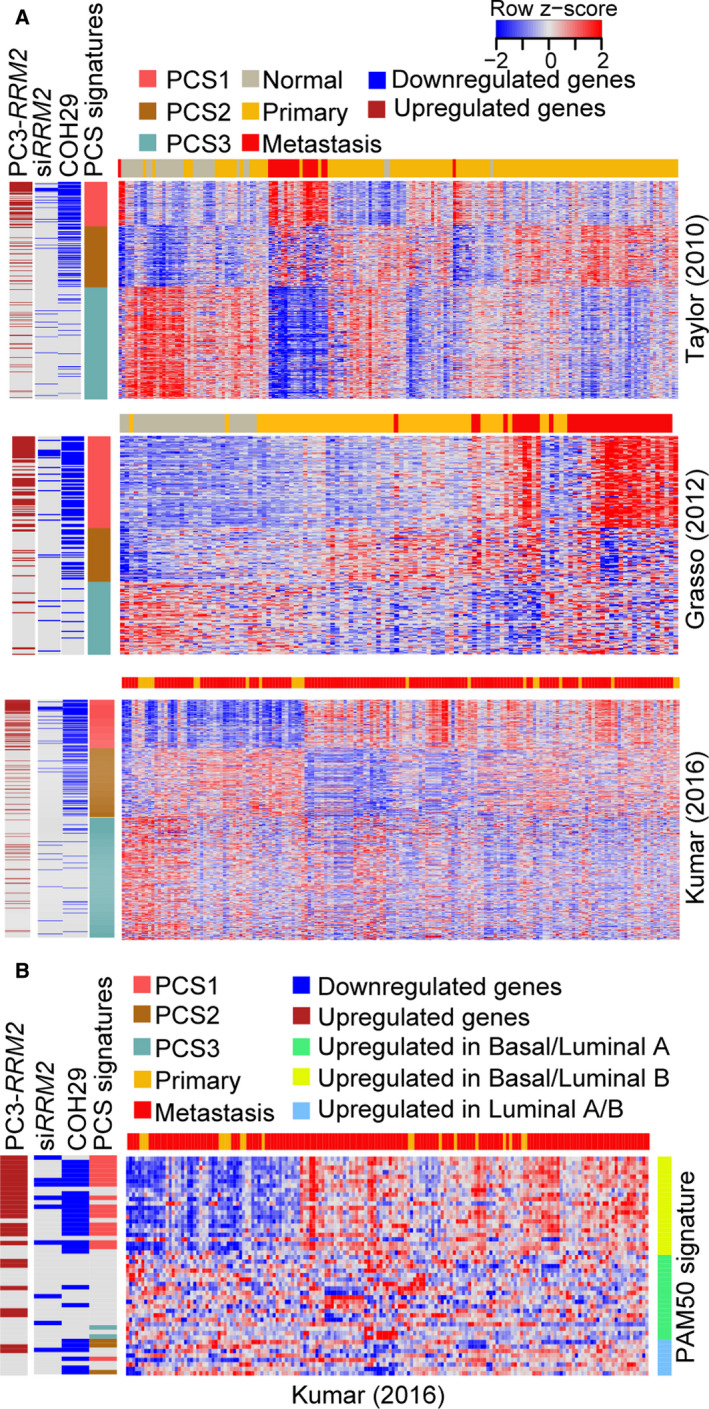
Inhibition of RRM2 specifically targets genes that define poor prognostic subtypes of prostate cancer. (A) Supervised hierarchical clustering of prostate cancer cases in the Taylor (top), Grasso (middle), and Kumar (bottom) cohorts, based on expression of PCS genes. Genes deregulated with *RRM2* overexpression (PC‐3‐*RRM2)* and inhibition of RRM2 (by COH29) are shown. (B) Supervised hierarchical clustering of prostate cancer cases from the Kumar cohort, based on expression of PAM50 classifier genes. Genes deregulated with *RRM2* overexpression (PC‐3‐*RRM2)* and inhibition of RRM2 (by COH29) are shown.

To further evaluate how the regulation of *RRM2* affects PCS signatures, we integrated our *RRM2*‐regulated transcriptome profiling from cell lines with gene expression data from prostate cancer clinical cohorts. We validated that the distinct gene profiling patterns of PCS genes correlated with tumor type in both the Taylor and Grasso (Grasso *et al*., 2012) cohorts (Fig. 5A). PCS1 genes were highly upregulated in metastatic tumors compared with normal prostate and primary tumors, PCS2 genes had high expression in primary tumors, and PCS3 genes were downregulated in prostate cancer compared with normal prostate. PCS genes also showed different profiling patterns in the Kumar cohort, which is mostly composed of metastatic cases (154/176). This suggests that PCS signatures not only distinguish normal, primary, and metastatic samples, but they may also define a subset of metastatic samples (Fig. 5A).

Prostate cancer subtype genes were compared to genes downregulated with RRM2 inhibition and genes upregulated with *RRM2* overexpression. Strikingly, COH29 treatment specifically inhibited the expression of most PCS1 genes and also targeted PCS2 genes (Fig. 5A). In addition to the PCS signatures, we also applied the PAM50 classifier in our analysis. In the Kumar cohort, we observed some separation of basal and luminal subtypes (Fig. 5B). The majority of PCS1 genes overlapped with genes upregulated in luminal B tumors; these cases have the poorest clinical prognoses (Zhao *et al*., 2017). Genes targeted by RRM2 inhibition or genes upregulated by *RRM2* overexpression in PC‐3 cells were highly enriched in luminal B genes (Fig. 5B). Together, these data suggest that RRM2 is a driver of the aggressive PCSs and that inhibition of RRM2 could specifically target the subtypes of prostate cancer with the worst prognosis.

### 3.7 The RRM2 signature may predict enzalutamide resistance in prostate cancer circulating tumor cells

Circulating tumor cells (CTCs) detach from the primary or secondary tumor sites and invade the bloodstream, and they have been reported to be useful prognostic biomarkers to aid prostate cancer diagnosis, treatment decision making, and patient follow‐up (Chung *et al*., 2019; De Laere *et al*., 2019; Nimir *et al*., 2019). The prognostic value of CTCs collected by the epithelial marker‐dependent method CellSearch has been established in the context of metastatic PC (Hegemann *et al*., 2016). Given the prognostic significance of the RRM2 signature in prostate cancer, we further investigated whether the RRM2 signature had clinical significance in prostate cancer CTCs.

**Fig. 6:**
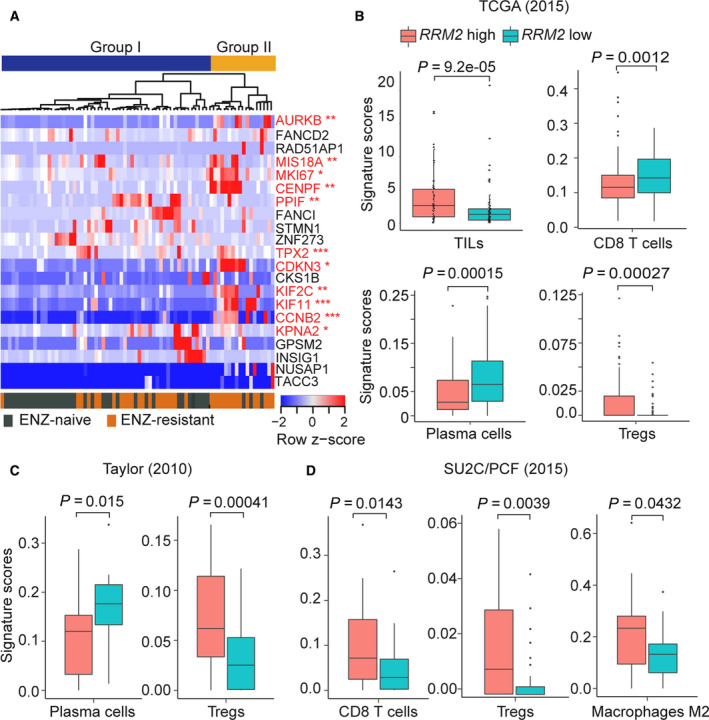
*RRM2* overexpression contributes to enzalutamide (ENZ) resistance and an immunosuppressive TIME. (A) Unsupervised hierarchical clustering of single‐cell RNA‐seq data from 77 CTCs from 13 patients with CRPC treated with enzalutamide (from GSE67980) based on expression of 21 genes from the RRM2 signature. Genes with expression that was significantly upregulated in the ENZ‐resistant CTCs of Group II are shown in red (11‐gene panel). *P < 0.05, **P < 0.01, ***P < 0.001. (B) Profiling of immune cells by CIBERSORT in the TCGA, (C) Taylor, and (D) SU2C/PCF cohorts. Significance was determined using Wilcoxon's rank‐sum test with Benjamini–Hochberg correction.

Based on single‐cell RNA‐seq data of CTCs from patients with CRPC, a 37‐gene panel was reported to identify patients with resistance to the AR antagonist enzalutamide (Miyamoto *et al*., 2015; You *et al*., 2016). As previously described (Miyamoto *et al*., 2015), patients who did not receive enzalutamide treatment before CTC collection were denoted as enzalutamide naïve, and patients whose cancer showed radiographic and/or PSA progression during enzalutamide therapy were denoted as enzalutamide‐resistant. We assessed whether our 126‐gene RRM2 signature could also predict enzalutamide resistance in CTCs. Of the 126‐gene signature, 21 genes were detectable in the CTC dataset (FDR < 0.05). Unsupervised hierarchical clustering based on expression of the 21 genes revealed two groups of CTCs (Fig. 6A). In Group I, 21 (36%) of the 59 CTCs were from enzalutamide‐resistant patients. In Group II, 15 (83%) of the 18 CTCs were from enzalutamide‐resistant patients. Increased expression of 11 of the 21 genes significantly correlated with enzalutamide resistance (Fig. 6A and Table S1); eight of the 11 genes were upregulated in the enzalutamide‐resistant CTCs of Group II (Fig. 6A). Surprisingly, only three genes of the 11‐gene panel from the RRM2 signature overlapped with the reported 37‐gene PCS panel (You *et al*., 2016). These results suggest that the 11‐gene panel derived from the RRM2 signature could be useful in predicting enzalutamide resistance in the CTCs of patients with CRPC. Furthermore, high expression of the 11‐gene signature is significantly associated with poor clinical outcomes (e.g., Gleason score and lethality in the Setlur cohort and DFS in the TCGA cohort; Fig. S1).

### 3.8 *RRM2* overexpression creates an immunosuppressive tumor‐immune microenvironment in prostate cancer

The tumor‐immune microenvironment (TIME), which can alter tumor progression and clearance, is affected by the genomic alterations of the tumor (Thorsson *et al*., 2018). We previously reported that overexpression of *RRM2* is highly correlated with copy number alteration (Mazzu *et al*., 2019); we therefore analyzed the correlation between *RRM2* overexpression and infiltration of immune cells in patients with prostate cancer from multiple cohorts. TIL scores were calculated in *RRM2*‐high and *RRM2*‐low groups from the TCGA cohort using deep‐learning models that integrate H&E staining of tissues (Saltz *et al*., 2018). Intriguingly, TIL enrichment was significantly greater in the *RRM2*‐high group than the *RRM2*‐low group (*P* = 9.2e‐05, Fig. 6B). To further examine the correlation between *RRM2* level and immune cell infiltration, we applied CIBERSORT analysis, a method of estimating the composition and abundance of immune cells from tumor biopsies (Newman *et al*., 2015). In the TCGA cohort, the infiltration of antitumor immune cells was significantly lower in the *RRM2*‐high group than in the *RRM2*‐low group (CD8^+^ T cells, *P* = 0.0012; plasma cells, *P* = 0.00015), whereas immunosuppressive regulatory T cells (Tregs) were more abundant in *RRM2*‐high tumors (*P* = 0.00027, Fig. 6B). Similarly, *RRM2*‐high tumors in the Taylor cohort, which is mostly composed of primary prostate cancer, had significantly fewer plasma cells (*P* = 0.015) and Tregs (*P* = 0.00041) than *RRM2*‐low tumors (Fig. 6C). In the SU2C/PCF cohort, which includes only metastatic CRPC, *RRM2*‐high tumors had significantly more immunosuppressive M2 macrophages (*P* = 0.0431) and Tregs (*P* = 0.0039) than *RRM2*‐low tumors (Fig. 6D). There was also greater infiltration of antitumor CD8^+^ T cells (*P* = 0.031) in *RRM2*‐high tumors. The signature scores of the 22 types of immune cells in the LM22 signature in the three prostate cancer cohorts are shown (Figs [Link], [Link], [Link]). Altogether, the high infiltration of immunosuppressive immune cells is suggestive of dysfunctional or exhausted cytotoxic T cells in *RRM2*‐high tumors.

## 4 Discussion

Molecular subtyping based on genomic alterations or oncogenic signatures has been successfully applied in multiple cancers. However, the heterogeneous nature of prostate cancer is a major impediment to developing a classification system with clinical relevance. Compared to individual biomarkers or other oncogenic signatures, the prostate cancer classification systems PCS and PAM50 are significantly better at identifying aggressive and resistant cases of prostate cancer.

The PCS classification system was developed and validated in 4600 samples from patients with prostate cancer. TCGA genomic subtypes (e.g., *ERG, ETV1/4, SPOP, FOXA1*, and others) were present across all the PCS categories (You *et al*., 2016). PCS1 is highly enriched with the SPOP subtype, whereas PCS2 tumors were overrepresented in ERG cancers (You *et al*., 2016). In the Genomic Resource Information Database (GRID) cohorts, PCS1 was enriched for Tomlins/ETS+ and Tomlins/SPINK1+ subtypes (You *et al*., 2016). Importantly, in the GRID cohorts, patients with PCS1 tumors had significantly shorter metastasis‐free survival than patients with PCS2 and PCS3 tumors, but no difference in metastatic progression was seen among the Tomlins categories (You *et al*., 2016). The PAM50 classification, which was developed using 3782 samples from patients with prostate cancer, was also recently shown to predict associations with clinical outcomes and response to treatment (Zhao *et al*., 2017).

This study expands upon our prior work on *RRM2* in prostate cancer and demonstrates that *RRM2* is a master driver of poor prognosis prostate cancer identified by both the PCS1 and PAM50 classification systems. RRM2 is essential for DNA synthesis and repair by producing dNTPs. Its level is rigorously regulated during the cell cycle, and delayed degradation may lead to genomic instability (D'Angiolella *et al*., 2012). *RRM2* is expressed at low levels in normal prostate tissue, but increased expression of *RRM2* is highly correlated with poor clinical outcomes in prostate cancer (Huang *et al*., 2014; Mazzu *et al*., 2019). We have previously demonstrated that *RRM2* is an oncogene in prostate cancer cells, regulates multiple oncogenic signaling pathways, and promotes EMT and angiogenesis (Mazzu *et al*., 2019). Although common pathways were activated by *RRM2* overexpression in LNCaP and PC‐3 cells, the majority of the upregulated genes were different (Fig. 2D–F), suggesting that *RRM2*‐regulated genes may be disease‐state‐specific.

Genomic alterations that occur in primary prostate cancer may not be enough to predict clinical behavior. The additional and distinct genomic alterations that develop over time add to the molecular heterogeneity of the primary disease and promote metastatic CRPC phenotypes. Therefore, it is not surprising that *RRM2* regulates distinct gene sets in two cell lines that may represent different disease states. LNCaP‐*RRM2* cells share a greater number of upregulated genes with the TCGA cohort, which only includes localized prostate cancer, than with the Kumar and SU2C/PCF cohorts, which mainly include metastatic CRPC (Fig. 2A). PC‐3 cells are more aggressive than LNCaP cells and may be more representative of advanced CRPC. This is supported by our data on the TCGA, Kumar, and SU2C/PCF cohorts, which demonstrates that a greater number of upregulated genes in PC‐3‐*RRM2* cells overlap with genes with expression that correlates with *RRM2* than LNCaP‐*RRM2* cells (126 genes in PC‐3; seven genes in LNCaP; Fig. 2B). These results further support the idea that *RRM2* may function by regulating distinct gene sets in different disease stages of prostate cancer.

Although FOXM1 was identified as a key regulator of the most aggressive subtype of prostate cancer (PCS1), it is difficult to target pharmacologically. Given that *RRM2*, a gene in the PCS1 and PAM50 signatures, has significant prognostic value in prostate cancer, we evaluated whether it could be another key regulator of aggressive subtypes. Intriguingly, expression of PCS1 genes is highly correlated with *RRM2* levels in prostate cancer cohorts; genes upregulated by *RRM2* overexpression in prostate cancer cells are also significantly enriched in the PCS1 signature. Furthermore, the 12 genes of the RRM2 signature that are also in the PCS1 and PAM50 signatures are luminal B genes. These results indicate that *RRM2* may be a master driver of the aggressive subtypes PCS1 and luminal B by directly or indirectly regulating the expression of critical genes. Ribonucleotide reductase inhibitors have been developed for cancer treatment (Knighton *et al*., 2018), and we previously reported the potency of the novel RRM2 inhibitor COH29 in prostate cancer (Mazzu *et al*., 2019). In this study, we confirmed that inhibiting RRM2 activity by siRNA or small molecule (COH29) specifically targets PCS1 and luminal B genes (Fig. 5).

Interestingly, the PAM50 classifier was recently reported as a pan‐carcinoma luminal/basal subtyping across epithelial tumors, and luminal B tumors were more sensitive to the ribonucleotide reductase inhibitor gemcitabine than the other subtypes (Zhao *et al*., 2019). Because gemcitabine‐induced amplification of *RRM2* is a mechanism of gemcitabine resistance (Duxbury *et al*., 2004; Zhou *et al*., 2001), we propose that RRM2‐specific inhibitors (e.g., COH29) may be more effective than gemcitabine for multiple epithelial cancers with similar luminal and basal subtypes. Additionally, we demonstrated that *RRM2* overexpression may contribute to AR antagonist resistance, suggesting that inhibition of RRM2 may delay the development of resistance.

Because the response rates to immunotherapy in prostate cancer are low, biomarkers are needed to determine which patients will respond. As a driver of aggressive prostate cancer, *RRM2* may have a major impact on the TIME. Here, we demonstrated that tumors with high expression of *RRM2* have more TILs, but the concomitant enrichment of immunosuppressive immune cells suggests that these TILs may be dysfunctional. Metastatic cases of prostate cancer with high *RRM2* levels have increased infiltration of immunosuppressive M2 macrophages, which may contribute to immune escape. It will be critical to validate the association between RRM2 overexpression and changes in the TIME by histologic staining in prostate cancer tissue. Patients with *RRM2*‐high tumors may be good candidates to receive immunotherapy because of increased TIL infiltration. Combination treatment of RRM2 inhibitors with immunomodulators to stimulate cytotoxic T cells and inhibit immunosuppressive cells may sensitize these tumors to immunotherapies.

## 5 Conclusions

In summary, we have shown that the genes shared by the PCS1 and luminal B signatures are regulated by *RRM2*. This suggests that *RRM2* is a master driver of aggressive PCSs. Targeting RRM2 may be an effective therapeutic option to reprogram the TIME and treat the subtypes of prostate cancer with poor prognosis.

## Conflict of interest

YZM has filed a patent application relevant to the work that is the subject of this paper: U.S. Provisional Patent Application No. 62/834914, RRM2 Signature as a Prognostic Marker for Prostate Cancer Survival, filed April 16, 2019; MSK Ref.: SK2019‐043‐01. As of January 29, 2020, PWK reports the following disclosures for the last 24‐month period: He has investment interest in Context Therapeutics LLC, DRGT, Placon, Seer Biosciences; is a company board member for Context Therapeutics LLC; and is a consultant/scientific advisory board member for Bavarian Nordic Immunotherapeutics, DRGT, GE Healthcare, Janssen, OncoCellMDX, Progenity, Seer Biosciences, and Tarveda Therapeutics; and serves on data safety monitoring boards for Genentech/Roche and Merck.

## Author contributions

YZM and PWK conceived and designed the study. YZM developed the methodology. YZM, JA, GC, LEJ, YY, MA, NK, NS, G‐SML, and PWK acquired the data, acquired and managed patient cohort data, and provided facilities. YZM, JA, SN, and PWK analyzed and interpreted the data such as statistical analysis, biostatistics, and computational analysis. YZM, JA, SN, and PWK wrote, reviewed, and/or revised the manuscript. YZM, JA, GC, YY, and LEJ involved in administrative, technical, or material support such as reporting or organizing the data. PWK and YZM supervised the data.

## Supporting information


**Fig. S1.** Clinical significance of 11‐gene signature in patient tissues.

**Fig. S2.** Profiling of immune cells in *RRM2*‐high and *RRM2*‐low prostate cancer samples from the TCGA cohort.

**Fig. S3.** Profiling of immune cells in *RRM2*‐high and *RRM2*‐low prostate cancer samples from the Taylor cohort.

**Fig. S4.** Profiling of immune cells in *RRM2*‐high and *RRM2*‐low prostate cancer samples from the SU2C/PCF cohort.

**Table S1.** Genes in the RRM2 signature and derived sub‐signatures.

Supplementary Material
