# Supplementary figures and images for: Ribonucleotide reductase small subunit M2 is a master driver of aggressive prostate cancer
Source: Mol Oncol. 2020 May 31;14(8):1881–97. doi: 10.1002/1878-0261.12706 (PMC7400792; doi:10.1002/1878-0261.12706)

# Figure S1

**A**

Setlur(2008)

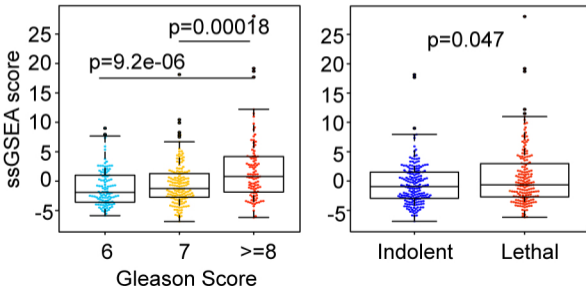**B**

TCGA cohort

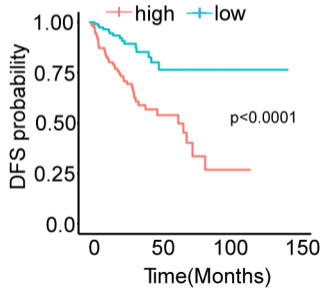

Supplement: Supplementary file 1 — Fig. S1. Clinical significance of 11‐gene signature in patient tissues. [file MOL2-14-1881-s001.pdf]

**Figure S2**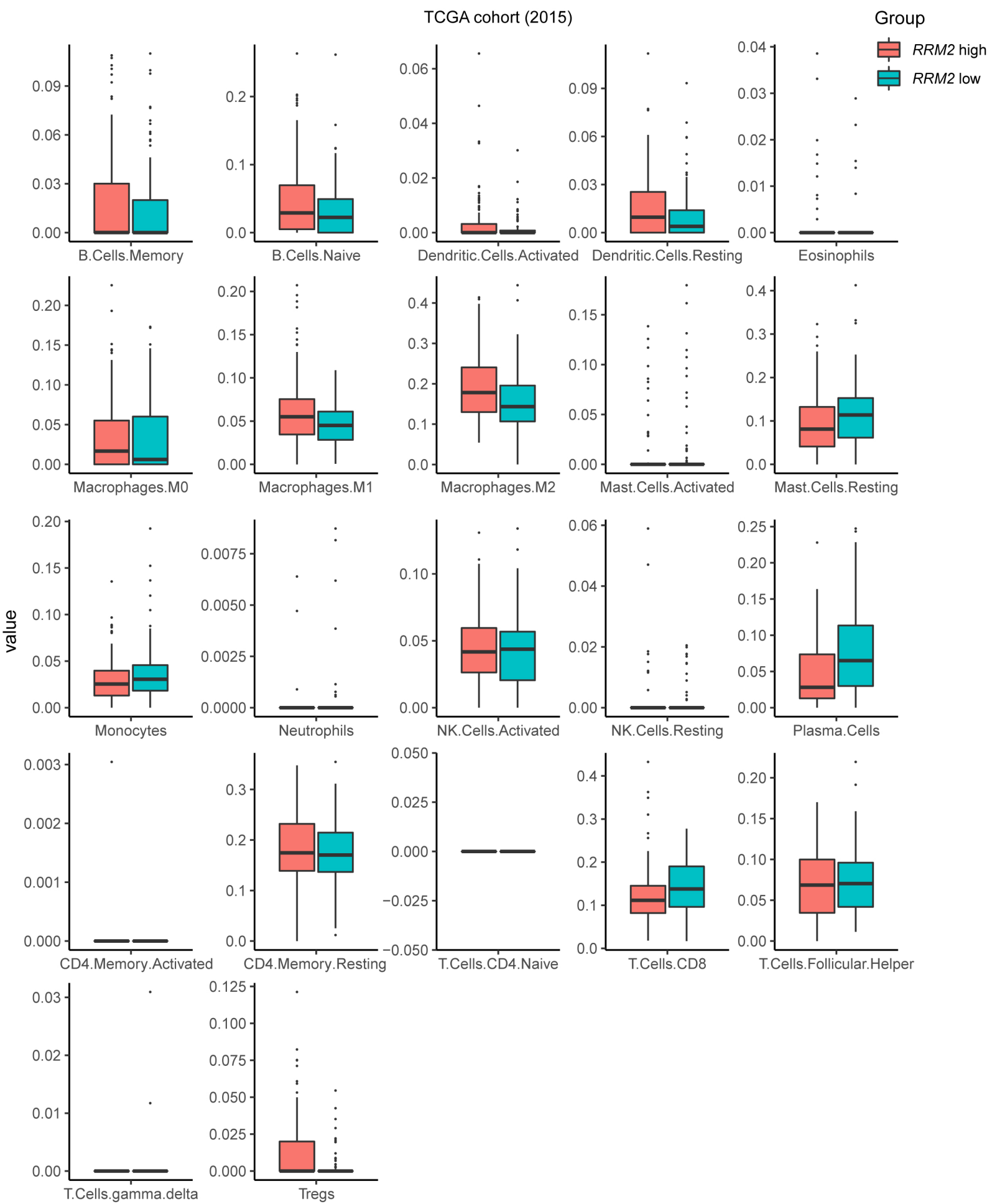

Supplement: Supplementary file 2 — Fig. S2. Profiling of immune cells in RRM2‐high and RRM2‐low prostate cancer samples from the TCGA cohort. [file MOL2-14-1881-s002.pdf]

**Figure S3**

Taylor cohort (2010)

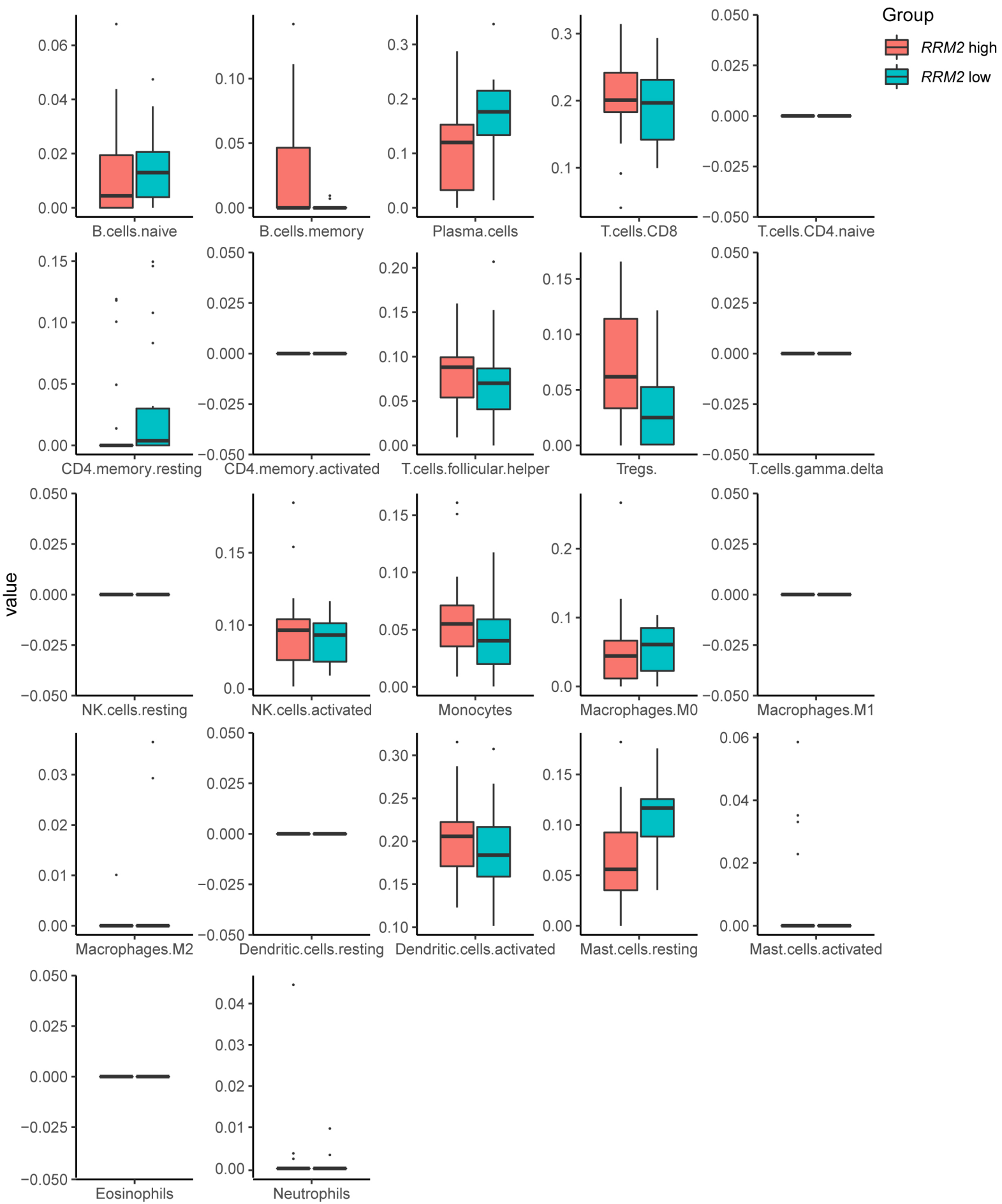

Supplement: Supplementary file 3 — Fig. S3. Profiling of immune cells in RRM2‐high and RRM2‐low prostate cancer samples from the Taylor cohort. [file MOL2-14-1881-s003.pdf]

**Figure S4**

SU2C/PCF cohort (2015)

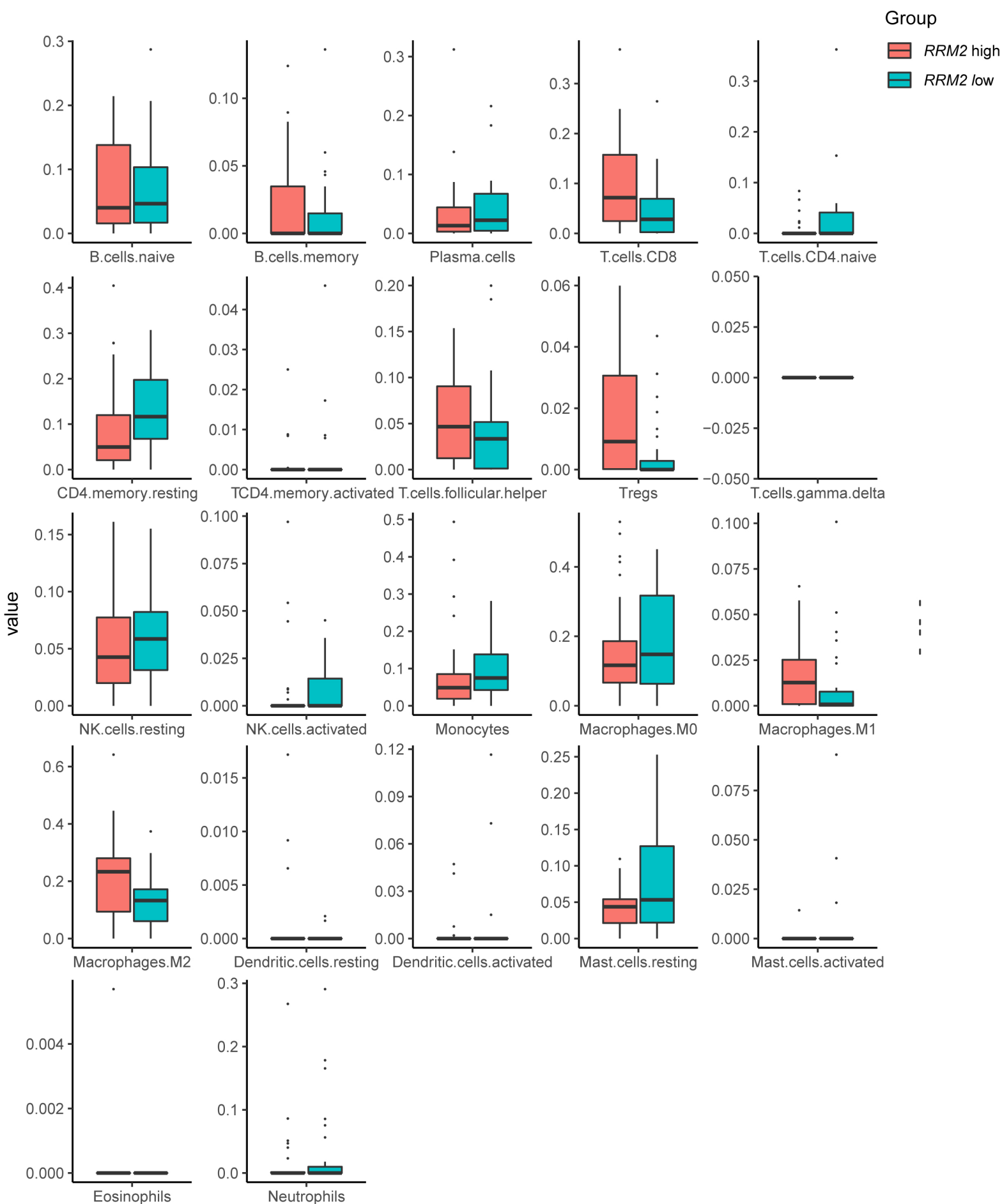

Supplement: Supplementary file 4 — Fig. S4. Profiling of immune cells in RRM2‐high and RRM2‐low prostate cancer samples from the SU2C/PCF cohort. [file MOL2-14-1881-s004.pdf]
