# Supplementary material for: Ribonucleotide reductase small subunit M2 is a master driver of aggressive prostate cancer
Source: Mol Oncol. 2020 May 31;14(8):1881–97. doi: 10.1002/1878-0261.12706 (PMC7400792; doi:10.1002/1878-0261.12706)
Supplement: Supplementary file 5 — Table S1. Genes in the RRM2 signature and derived sub‐signatures. [file MOL2-14-1881-s005.pdf]

Table S1. Genes in the RRM2 signature and derived sub-signatures. The list of genes that comprise each signature.

**RRM2 signature** [a]		**RRM2/PCS1** [b]	**PCS1/PAM50**[c]	**RRM2/PCS1/ PAM50** [d]	**RRM2/CTCs**[e]
**126-gene panel**		**50-gene panel**	**14-gene panel**	**12-gene panel**	**11-gene panel**
ANLN	*KIF18B*	*ANLN*	*ANLN*	*ANLN*	*AURKB*
ARL6IP1	*KIF20A*	*AURKA*	*BIRC5*	*BIRC5*	*CCNB2*
ASF1B	*KIF20B*	*BIRC5*	*CCNB1*	*CCNB1*	*CDKN3*
ATAD2	*KIF23*	*BUB1*	*CDC20*	*CDC20*	*CENPF*
ATAD5	*KIF2C*	*BUB1B*	*CDC6*	*CDC6*	*FANCI*
AUNIP	*KIF4A*	*CCNA2*	*CENPF*	*CENPF*	*KIF11*
AURKA	*KPNA2*	*CCNB1*	*CEP55*	*CEP55*	*KIF2C*
AURKB	*MAD2L1*	*CCNB2*	*KIF2C*	*KIF2C*	*MIS18A*
BIRC5	*MELK*	*CDC20*	*MELK*	*MELK*	*MKI67*
BLM	*MIS18A*	*CDC6*	*MKI67*	*MKI67*	*PPIF*
BORA	*MIS18BP1*	*CDCA5*	*PTTG1*	*PTTG1*	*TPX2*
BUB1	*MKI67*	*CDCA8*	*TYMS*	*UBE2T*	
BUB1B	*MNS1*	*CDK1*	*UBE2C*		
CCNA2	*MYBL2*	*CDKN3*	*UBE2T*		
CCNB1	*NCAPG*	*CENPE*			
CCNB2	*NCAPG2*	*CENPF*			
CCNE1	*NCAPH*	*CENPM*			
CDC20	*NUF2*	*CEP55*			
CDC6	*NUSAP1*	*CIT*			
CDCA3	*ORC1*	*CKS2*			
CDCA5	*PARP2*	*EZH2*			
CDCA8	*PBK*	*HJURP*			
CDK1	*PCLAF*	*HMGB2*			
CDKN3	*PIF1*	*HMMR*			
CDT1	*PIGA*	*KIF11*			
CENPE	*PIMREG*	*KIF15*			
CENPF	*PKMYT1*	*KIF20A*			
CENPH	*PLK1*	*KIF23*			
CENPM	*PLK4*	*KIF2C*			
CENPP	*POLQ*	*KIF4A*			
CENPU	*POT1*	*MELK*			
CEP152	*PPIF*	*MKI67*			
CEP55	*PRC1*	*NCAPG*			
CIT	*PSMC3IP*	*NCAPG2*			
CKAP2	*PSRC1*	*NUF2*			
CKAP5	*PTTG1*	*NUSAP1*			
CKS1B	*RAD51*	*PBK*			
CKS2	*RAD51AP1*	*PKMYT1*			
CTSV	*RAD54B*	*POLQ*			
DBF4	*RAD54L*	*PRC1*			
DDIAS	*RFC3*	*PTTG1*			
DEPDC1	*RMI2*	*RAD54L*			
DEPDC1B	*SHCBP1*	*SPAG5*			
DNA2	*SKA1*	*STMN1*			
DNMT3B	*SKA3*	*TACC3*			
DONSON	*SPAG5*	*TK1*			
DSCC1	*SPC25*	*TOP2A*			
EZH2	*STIL*	*TPX2*			
FAM72D	*STMN1*	*UBE2T*			
FAM83D	*TACC3*	*ZWINT*			
FANCD2	*TK1*				
FANCI	*TOP2A*				
GINS1	*TPX2*				
GINS2	*TRIM59*				
GPC2	*TUBA1B*				
GPSM2	*UBE2S*				
GTSE1	*UBE2T*				
HJURP	*WDR62*				
HMGB2	*WEE1*				
HMMR	*ZGRF1*				
INSIG1	*ZNF273*				
KIF11	*ZNF695*				
KIF15	*ZWINT*				

a. The RRM2 signature consists of 126 genes. It was created by intersecting upregulated genes in PC-3-*RRM2* cells with genes with expression that was positively correlated with RRM2 overexpression in The Cancer Genome Atlas, Kumar, and Stand Up To Cancer/Prostate Cancer Foundation cohorts.
b. 50-gene panel consists of common genes between the RRM2 signature and the PCS1 signature genes.
c. 14-gene panel consists of genes shared by PCS1 subtype and PAM50 classifiers.
d. 12-gene panel consists of common genes in the RRM2 signature, PCS1 signature, and PAM50 classifier.
e. 11-gene panel consists of genes in the RRM2 signature and those upregulated in enzalutamide-resistant circulating tumor cells (CTCs). CTC expression data derived from single-cell RNA-seq profiling of CTCs from patients with prostate cancer (GEO: GSE67980).
