# Supplementary material for: Ribonucleotide reductase small subunit M2 is a master driver of aggressive prostate cancer
Source: Mol Oncol. 2020 May 31;14(8):1881–97. doi: 10.1002/1878-0261.12706 (PMC7400792; doi:10.1002/1878-0261.12706)
Supplement: Supplementary file 6 — Supplementary Material [file MOL2-14-1881-s006.pdf]

Supplemental Figures

Fig. S1. Clinical significance of 11-gene signature in patient tissues. (A) Association between expression of the 11-gene signature with Gleason score (left) and lethality (right) in the Setlur cohort. (B) Clinical significance of 11-gene signature expression in the TCGA cohort. Samples were ranked based on expression of the 12-gene sub-signature, and Kaplan-Meier curves were used to estimate survival differences between patients in the top and bottom 25th percentiles of expression. The log-rank test was calculated to determine significance.

Fig. S2. Profiling of immune cells in *RRM2*-high and *RRM2*-low prostate cancer samples from the TCGA cohort. *RRM2* expression was used to categorize cases as *RRM2* high (upper quantile) and *RRM2* low (lower quantile), and CIBERSORT was used to profile immune cell populations using the LM22 signature. Significance was determined using Wilcoxon's rank sum test with Benjamini-Hochberg correction.

Fig. S3. Profiling of immune cells in *RRM2*-high and *RRM2*-low prostate cancer samples from the Taylor cohort. *RRM2* expression was used to categorize cases as *RRM2* high (upper quantile) and *RRM2* low (lower quantile), and CIBERSORT was used to profile immune cell populations using the LM22 signature. Significance was determined using Wilcoxon's rank sum test with Benjamini-Hochberg correction.

Fig. S4. Profiling of immune cells in *RRM2*-high and *RRM2*-low prostate cancer samples from the SU2C/PCF cohort. *RRM2* expression was used to categorize cases as *RRM2* high (upper quantile) and *RRM2* low (lower quantile), and CIBERSORT was used to profile immune cell populations using the LM22 signature. Significance was determined using Wilcoxon's rank sum test with Benjamini-Hochberg correction.
